# Experimentally Manipulating Items Informs on the (Limited) Construct and Criterion Validity of the Humor Styles Questionnaire

**DOI:** 10.3389/fpsyg.2017.00616

**Published:** 2017-04-20

**Authors:** Willibald Ruch, Sonja Heintz

**Affiliations:** Personality and Assessment, Department of Psychology, University of ZurichZurich, Switzerland

**Keywords:** Humor Styles Questionnaire, humor, measurement, validity, item wording, well-being, personality, scale construction

## Abstract

How strongly does humor (i.e., the construct-relevant content) in the Humor Styles Questionnaire (HSQ; Martin et al., 2003) determine the responses to this measure (i.e., construct validity)? Also, how much does humor influence the relationships of the four HSQ scales, namely affiliative, self-enhancing, aggressive, and self-defeating, with personality traits and subjective well-being (i.e., criterion validity)? The present paper answers these two questions by experimentally manipulating the 32 items of the HSQ to only (or mostly) contain humor (i.e., construct-relevant content) or to substitute the humor content with non-humorous alternatives (i.e., only assessing construct-irrelevant context). Study 1 (*N* = 187) showed that the HSQ affiliative scale was mainly determined by humor, self-enhancing and aggressive were determined by both humor and non-humorous context, and self-defeating was primarily determined by the context. This suggests that humor is not the primary source of the variance in three of the HQS scales, thereby limiting their construct validity. Study 2 (*N* = 261) showed that the relationships of the HSQ scales to the Big Five personality traits and subjective well-being (positive affect, negative affect, and life satisfaction) were consistently reduced (personality) or vanished (subjective well-being) when the non-humorous contexts in the HSQ items were controlled for. For the HSQ self-defeating scale, the pattern of relationships to personality was also altered, supporting an positive rather than a negative view of the humor in this humor style. The present findings thus call for a reevaluation of the role that humor plays in the HSQ (construct validity) and in the relationships to personality and well-being (criterion validity).

## Introduction

Most questionnaire items contain both the construct they intend to measure (i.e., the construct-relevant content) but also additional information, which should measure the relevant content in a variety of circumstances to increase its representativeness (see Epstein, 1983). In a homogenous scale (i.e., a scale that uniformly measures a single construct), one would thus expect similar contents, as these form the core of the scale, but somewhat dissimilar contexts. For example, the construct of “liking to laugh” can be shown in different contexts, such as being with family or friends, being told a joke, or watching a funny movie in the cinema. The tendency to laugh more than others should then generalize across the different situations. The item contexts should vary so that summing up the items over a scale strengthens the variance due to the core content and more or less averages out the different situations. This additional information might not only refer to situational contexts, but also to states, feelings, or evaluations that specify the core content in more detail. Importantly, mostly the variance contributed by the content should be the relevant one.

Besides the core content, additional elements may unintentionally produce a considerable amount of variance in a scale if it is homogenous and strongly represented or if the content is not that salient. For example, measuring “liking to laugh” with items such as “While I deliver a lecture to my class I laugh a lot,” “When my colleagues make a funny remark in a faculty meeting I laugh easily,” and “My assistants and I laugh a lot when we hear that our article was accepted.” The answers to these items might not be determined by the tendency of liking to laugh alone, and relationships to other constructs (e.g., vocational background) would likely be biased by the additional elements in the items. Messick's (1995) mentioned this “construct-irrelevant variance” as a threat to construct validity, which occurs if “the assessment is too broad, containing excess reliable variance associated with other distinct constructs” (p. 742). Hence the amount of variance contributed by the construct-relevant content and the non-relevant context can be an indicator of the construct validity of an instrument, as the scale compositions and their relations to other constructs should be mainly driven by the construct they are intended to measure (i.e., construct-relevant variance), and less so or not all by the remainder of the item (i.e., construct-irrelevant variance).

How can the contribution of construct-relevant and construct-irrelevant variance be empirically investigated? For example, the item wording could be experimentally altered to only assess construct-relevant contents in the items, or the relevant content could be removed to yield purely construct-irrelevant items. Although not investigating construct validity, Haigler and Widiger (2001) experimentally manipulated the items of the NEO-PI-R to reverse their desirability/adaptiveness without changing the item content itself. Specifically, they changed the items from desirable/adaptive to undesirable/maladaptive, or from having a positive to a negative connotation. They simply added descriptors such as “too much” or “excessively” to the items, resulting in a reversal of desirability/adaptiveness as judged by raters. In addition, the pattern of correlations with personality disorders changed for the rephrased items in a sample of 86 adult outpatients. Most strikingly, the experimentally manipulated version of conscientiousness correlated strongly and positively with obsessive-compulsive personality, and agreeableness correlated strongly with dependent and avoidant personality disorders (while the original NEOPI-R scales showed mostly zero correlations). This study empirically supports the idea that already slight changes in item wording can change the construct that is measured (which was also found in a recent study by Blasberg et al., 2016) and its desirability/adaptiveness.

The present paper combines both Messick's (1995) ideas about construct-irrelevant variance in the contexts and the experimental manipulations of item wordings. We aim at experimentally disentangling the construct-relevant content from the remainder of the item by creating new items that only assess the core content (i.e., pure construct-relevant indicators) or by replacing the core content (i.e., pure construct-irrelevant indicators). The first study compares the similarities of the two experimentally manipulated versions with the original items and scales to yield insights into the construct validity of the original instrument. To support construct validity, relationships of the original version should be higher with the construct-relevant indicators than with the construct-irrelevant ones. Ideally, each original scale should perfectly converge with its pure construct-relevant indicators, supporting that it only assesses the construct to be measured and not other unrelated and possibly confounding elements. The second study extends the item wording manipulation to test the criterion validity of a scale. Controlling for the construct-irrelevant indicators (using the experimentally rephrased items) should reveal the “pure” correlations of the constructs under question with a set of external criteria.

This procedure is applied to the Humor Styles Questionnaire items (HSQ; Martin et al., 2003), which assesses four humor styles that represent functions of humor in everyday life, and especially those functions relevant to psychosocial well-being. The construct-relevant content hence comprises humor (including joking, laughing, and making fun of oneself and others) and functions (using humor to enhance oneself or relationships to others). The four humor styles are affiliative (amusing others, liking to laugh, and making jokes to enhance one's relationships with others), self-enhancing (amusing oneself and cheering oneself up with humor to enhance oneself), aggressive (making jokes, laughing at others, and teasing others to enhance oneself), and self-defeating (making fun of oneself and letting others laugh about oneself to enhance one's relationships with others). According to Martin et al. (2003), the affiliative humor style should be associated with better psychosocial well-being (as it should be affirming of both self and others). The self-enhancing humor style should be associated with better psychological well-being (as it entails a coping aspect). The aggressive humor style should be associated with lower social well-being (as it entails putting others down). Finally, the self-defeating humor style should be associated with lower psychological well-being (due to a negative self-evaluation and emotional avoidance underlying it).

The present investigation focuses on the humor-related contents, as the role humor plays in the HSQ is of special interest: First, the HSQ is the most widely used questionnaire in research on individual differences in humor (see Martin, 2015). Second, its interpretations usually focus on the humor-related content, for example, considering humor as a mediator in the relationship with well-being or implementing humor exercises based on findings with the HSQ. Third and foremost, inspection of its items frequently shows a salient context where it does not deem necessary (e.g., “being alone” in self-enhancing humor items; laughing at oneself “too much” in self-defeating humor). It seems necessary to demonstrate empirically that these variations in context do average out and do not bias the overall meaning of the scale. Thus, investigating to what extent the four HSQ scales and their relationships to relevant criteria (in this case subjective well-being) are determined by humor vs. other construct-irrelevant elements is an important indicator of their construct and criterion validity.

The experimental manipulation of the 32 items of the HSQ proceeded as follows: They were rephrased to only contain their construct-relevant content (i.e., humor-related words or phrases; “*Humor-HSQ*”) or the construct-relevant content was replaced (“*No-Humor-HSQ*”). To generate the No-Humor-HSQ, the items were minimally changed to replace the humor elements (substituting them by something similar but non-humorous). To generate the Humor-HSQ, everything that went beyond the humor content (be it situational conditions, thoughts or feelings during the humor behavior, or evaluations of the behavior) was stripped of. For example, the HSQ self-defeating item “I let people laugh at me or make fun at my expense more than I should” can be reduced to its humor part (“I let people laugh at me or make fun at my expense”) or the humor content can be replaced (“I let people offend me or look down on me more than I should”). The former reduces the humor-related constructs to their core and the latter leaves the item intact but eliminates the reference to humor (i.e., leaves only construct-irrelevant context).

## Study 1: composition of the humor styles questionnaire

Study 1 tests the construct validity of the HSQ by comparing the original HSQ with the Humor-HSQ and the No-Humor-HSQ. First, it is expected that the internal consistency of the three HSQ versions will vary in a predictable way. To the extent that the non-humorous elements produce variance, it makes the items more dissimilar, thereby increasing the internal consistencies of the Humor-HSQ scales and reducing the internal consistencies of the No-Humor-HSQ scales (in comparison to the HSQ scales). Second, the intercorrelations of the three HSQ versions should be influenced similarly. Ideally, if the non-humorous elements produce only construct-irrelevant variance that is averaged out within the four scales, then the HSQ should not correlate (or only slightly) with No-Humor-HSQ scales, and high with the Humor-HSQ (approaching unity in true-score correlations). The more construct-relevant variance is contributed by the non-humorous elements, the higher correlations can be expected between the No-Humor-HSQ and the HSQ, and the lower correlations can be expected between the Humor-HSQ and the HSQ.

### Materials and methods

#### Participants

Of the 289 German-speaking participants who started the survey, 201 (69.9%) completed all the items. A total of 187 participants (17.1% men) with a median age of 24 (*M* = 28.81, *SD* = 10.76) ranging from 17 to 63 years provided valid responses in this study (14 participants were excluded because they answered more than 12 items per minute, indicating inattentiveness)^1^. Participants were primarily Swiss (58.3%), German (34.2%), and from several other nations. Most participants were well-educated, with 34.2% being college or university students, 33.2% having passed tertiary education, 24.1% having A-levels, and 7.0% having an apprenticeship. A subsample of the present data was used by Ruch and Heintz (2013, study 2). None of the present results have been published before and they extend the previous study by investigating the overlap between the three HSQ versions.

#### Instruments

##### Humor styles questionnaire (HSQ; Martin et al., 2003; German version by Ruch and Heintz, 2016)

The HSQ consists of 32 items measuring the four humor styles. Sample items are “I don't often joke around with my friends.” (affiliative, negatively keyed), “Even when I'm by myself, I'm often amused by the absurdities of life” (self-enhancing), “If someone makes a mistake, I will often tease them about it.” (aggressive), and “I let people laugh at me or make fun at my expense more than I should” (self-defeating). The instrument employs a seven-point Likert scale from “totally disagree” (1) to “totally agree” (7).

##### Content version derived from the HSQ (humor-HSQ)

The 32 HSQ items were rephrased to only capture the relevant humor content, resulting in four humor scales. Sample items are “I don't often joke around” (affiliative, negatively keyed), “I'm often amused by the absurdities of life.” (self-enhancing), “I often tease others” (aggressive), and “I let people laugh at me or make fun at my expense.” (self-defeating). The instrument employs the same Likert scale as the HSQ. The item order and keying of the original HSQ was preserved except for one self-enhancing and one aggressive item, which were positively keyed to ensure comprehensibility.

Two raters (the second author and a graduate psychology student) judged which parts of the HSQ items referred to humor vs. context. Interrater agreement (Cohen's kappa) was 0.77. Only the parts judged as containing humor were retained for the Humor-HSQ items (e.g., the item “Even when I'm by myself, I'm often amused by the absurdities of life” was rephrased into “I'm often amused by the absurdities of life”). The set of items was finalized in a discussion between the two authors.

##### Context version derived from the HSQ (No-Humor-HSQ)

The 32 HSQ items were rephrased to only capture the relevant context component, resulting in four humor-free context scales. Sample items are “I don't often converse with my friends” (affiliative, negatively keyed), “Even when I'm by myself, I often occupy myself with the little things in life.” (self-enhancing), “If someone makes a mistake, I will often reproach them about it.” (aggressive), and “I let people offend me or look down on me more than I should” (self-defeating). The instrument employs the same Likert scale, item order and keying as the HSQ. The 32 items of the Humor-HSQ and the No-Humor-HSQ are listed in the Table A1 in Appendix.

The item rephrasing process for the No-Humor-HSQ proceeded in two steps: (a) Identifying the humorous word(s) or expression(s) in each HSQ item, and (b) substituting it/them with a non-humorous, but equivalent counterpart. In step (a), the two raters judged the core humor word(s) of each item (Cohen's kappa = 0.82). In addition, every humor word that was not agreed upon (e.g., “blunder”) was further analyzed using two online-thesauri (www.openthesaurus.de and www.thesaurus.com) to ensure that either the definition or one of the synonyms related to humor.

After all humorous words had been identified, they were substituted in step (b) with a non-humorous expression that was as equivalent as possible (e.g., “misapprehension” instead of “blunder,” “enthralling,” or “beautiful” instead of “funny”). The criterion of being humor-free was fulfilled if none of the meanings and synonyms contained a humorous word (using the two online thesauri). Equivalent meant that the word was from the same part of speech (e.g., verb, adjective, noun) and encompassed a similar level of activity (e.g., communication, action) and affect (e.g., positive, negative). In addition, nine raters (post-graduate psychologists) judged the 32 newly written No-Humor-HSQ items for their humor content (*Does the item still contain a trace/hint to humor?*), similarity (*Is/Are the replaced “humor-free” word[s] similar to the original one[s], or is there any deviation in relation to part of speech, activity, or affect?*), and overall meaningfulness (*Is the item still meaningful or are there any inconsistencies that hamper or prevent understanding the item?*). Items were iteratively improved according to each rater's judgments, and the set of items was then finalized in a discussion between the two authors to ensure that the No-Humor-HSQ items did not contain humor, that they were similar to the original, and that they were meaningful.

#### Procedure

The data were collected in an online survey (www.unipark.info) employing a forced-choice item format. The No-Humor-HSQ was presented first, followed by the Humor-HSQ and then the original HSQ. Further variables on personality and well-being were collected that are not relevant to the present study, yet they were used as “fillers” in between the three HSQ versions. Participants were recruited via several means, including mailing lists of the University of Zurich, social media platforms, and bulletins. They were offered a personalized feedback and/or course credit in psychology for their participation. The study was conducted in compliance with the local ethical guidelines and participants provided online informed consent.

#### Data analysis

First, internal consistencies (McDonald's omega) and scale intercorrelations were computed to compare the three versions of the HSQ (original, humor, and no-humor). McDonald's omega was computed with the MBESS package (Kelley and Lai, 2012) in R (R Core Team, 2016). The differences between the (dependent) correlations were compared using the psych package (Revelle, 2015) in *R*. Correction for attenuation [according to Spearman's (1904) classical formula] was employed to reveal the true-score correlations between the scales of the three HSQ versions.

### Results

#### Observed scale intercorrelations

Table 1 shows the means, standard deviations, intercorrelations, and internal consistencies of the HSQ, Humor-HSQ, and No-Humor-HSQ scales.

**Table 1:** **Means, standard deviations, intercorrelations, and internal consistencies of the Humor Styles Questionnaire (HSQ) scales and the derived Humor-HSQ and No-Humor-HSQ scales**.

	***M***	***SD***	**(1)**	**(2)**	**(3)**	**(4)**	**(5)**	**(6)**	**(7)**	**(8)**	**(9)**	**(10)**	**(11)**	**(12)**
**HSQ**														
(1) AF	39.98	9.81	*0.91*											
(2) SE	34.97	8.58	0.40^*^	*0.87*										
(3) AG	26.93	6.90	0.25^*^	−0.12	*0.74*									
(4) SD	24.86	8.39	−0.05	−0.16^*^	0.21^*^	*0.88*								
**HUMOR-HSQ**														
(5) AF	39.14	10.47	0.91^*^	0.40^*^	0.29^*^	0.02	*0.94*							
(6) SE	37.69	8.26	0.61^*^	0.83^*^	0.07	−0.10	0.65^*^	*0.89*						
(7) AG	31.57	7.89	0.53^*^	0.07	0.77^*^	0.28^*^	0.61^*^	0.33^*^	*0.80*					
(8) SD	31.91	8.87	0.51^*^	0.23^*^	0.31^*^	0.61^*^	0.57^*^	0.43^*^	0.58^*^	*0.89*				
**NO-HUMOR HSQ**														
(9) AF	36.73	9.07	0.74^*^	0.42^*^	0.10	−0.25^*^	0.67^*^	0.57^*^	0.29^*^	0.30^*^	*0.84*			
(10) SE	35.57	7.08	0.20^*^	0.75^*^	−0.24^*^	−0.20^*^	0.18^*^	0.57^*^	−0.10	0.11	0.35^*^	*0.73*		
(11) AG	27.74	5.50	0.27^*^	−0.04	0.76^*^	0.01	0.29^*^	0.08	0.59^*^	0.17^*^	0.19^*^	−0.19^*^	*0.42*	
(12) SD	26.50	7.39	−0.23^*^	−0.32^*^	0.12	0.76^*^	−0.15^*^	−0.26^*^	0.11	0.30^*^	−0.37^*^	−0.33^*^	−0.02	*0.72*

*N = 187. AF, affiliative; SE, self-enhancing; AG, aggressive; SD, self-defeating. McDonald's omegas in italics. Theoretical minimum mean of the scales = 8, maximum mean = 56*.

* *p < 0.05*.

As shown in Table 1, the internal consistencies of the Humor-HSQ scales were high (≥0.80) and always numerically higher than the ones of the homologous HSQ scales. In turn, the internal consistencies of the No-humor-HSQ scales were always numerically lower than the HSQ scales, yet they still evidenced good internal consistencies (>0.70), with the exception of the aggressive scale (0.42). The correlations among the scales of the Humor- and No-Humor-HSQ with the homologous HSQ scales were all high (*r*s ≥ 0.61, *p*s < 0.05), indicating that both the humor and the non-humor elements were relevant for the HSQ scales. Comparing the size of the correlations between the homologous scales of the two HSQ versions with the original HSQ, significant differences were found for the affiliative (*t* = 7.03, *p* < 0.001), self-enhancing (*t* = 2.42, *p* = 0.017), and self-defeating (*t* = −2.91, *p* = 0.004) scales. The correlations of the HSQ affiliative and self-enhancing scales were significantly larger with the Humor-HSQ than with the No-Humor-HSQ, indicating that the humor content was more relevant for these HSQ scales than the non-humorous elements. This effect was reversed for the HSQ self-defeating scale; that is, the No-Humor-HSQ, in comparison to the Humor-HSQ, correlated significantly higher with the HSQ. This indicates that the non-humorous elements were more important in the HSQ self-defeating scale than its humor core.

Numerically comparing the scale intercorrelations within each HSQ version, a few peculiarities can be noted. First, the HSQ and the No-Humor-HSQ showed small to medium intercorrelations (both positive and negative), while the Humor-HSQ scales were all positively correlated (medium to large effects). Second, the HSQ affiliative scale had large intercorrelations with all Humor-HSQ scales. Third, the Humor-HSQ self-defeating scale correlated positively with all HSQ scales (small to large effects), including the HSQ self-enhancing scale.

#### True-score scale intercorrelations

The true-score correlations [using a double correction for attenuation with Spearman's (1904) formula] were close to one for three of the four HSQ and Humor-HSQ scales: Affiliative (0.98), self-enhancing (0.94), and aggressive (1.00), while the value was considerably lower for self-defeating (0.69). However, correlations were also close to one for three of the four HSQ and No-Humor-HSQ scales: Self-enhancing (0.94), aggressive (1.00) and self-defeating (0.95), while the true-score correlation was slightly lower for affiliative (0.85).

#### Item intercorrelations

This raises the question to what extent the findings at the scale-level are also present at the level of the individual items. As each item was assessed in all three versions of the HSQ, comparing their correlations with one another can reveal the relative influence of humor and non-humor elements within each item. Table 2 shows the intercorrelations of the HSQ items with the corresponding items of the Humor-HSQ and the No-Humor-HSQ.

**Table 2:** **Intercorrelations of the Humor Styles Questionnaire (HSQ) Items with the Homologous Items of the Humor-HSQ and No-Humor-HSQ**.

	**AF**	**SE**	**AG**	**SD**
**ITEM 1**				
HSQ with Humor-HSQ	0.73^*^^a^	0.67^*^	0.48^*^	0.42^*^
HSQ with No-Humor HSQ	0.49^*^^b^	0.63^*^	0.55^*^	0.41^*^
Humor-HSQ with No-Humor HSQ	0.46^*^	0.39^*^	0.20^*^	0.08
**ITEM 2**				
HSQ with Humor-HSQ	0.85^*^^a^	0.75^*^^a^	0.46^*^	0.65^*^
HSQ with No-Humor HSQ	0.49^*^^b^	0.03^b^	0.53^*^	0.59^*^
Humor-HSQ with No-Humor HSQ	0.42^*^	−0.07	0.36^*^	0.51^*^
**ITEM 3**				
HSQ with Humor-HSQ	0.48^*^	0.50^*^	0.24^*^^a^	0.50^*^
HSQ with No-Humor HSQ	0.42^*^	0.61^*^	0.53^*^^b^	0.59^*^
Humor-HSQ with No-Humor HSQ	0.39^*^	0.39^*^	0.14^*^	0.32^*^
**ITEM 4**				
HSQ with Humor-HSQ	0.79^*^^a^	0.67^*^	0.60^*^	0.30^*^
HSQ with No-Humor HSQ	0.64^*^^b^	0.68^*^	0.59^*^	0.19^*^
Humor-HSQ with No-Humor HSQ	0.61^*^	0.56^*^	0.32^*^	0.08
**ITEM 5**				
HSQ with Humor-HSQ	0.67^*^^a^	0.65^*^	0.43^*^^a^	0.43^*^^a^
HSQ with No-Humor HSQ	0.46^*^^b^	0.72^*^	0.73^*^^b^	0.67^*^^b^
Humor-HSQ with No-Humor HSQ	0.43^*^	0.51^*^	0.31^*^	0.26^*^
**ITEM 6**				
HSQ with Humor-HSQ	0.77^*^^a^	0.54^*^^a^	0.60^*^	0.48^*^^a^
HSQ with No-Humor HSQ	0.44^*^^b^	0.30^*^^b^	0.60^*^	0.71^*^^b^
Humor-HSQ with No-Humor HSQ	0.35^*^	0.35^*^	0.58^*^	0.29^*^
**ITEM 7**				
HSQ with Humor-HSQ	0.62^*^^a^	0.46^*^^a^	0.53^*^^a^	0.05^a^
HSQ with No-Humor HSQ	0.35^*^^b^	0.67^*^^b^	0.66^*^^b^	0.73^*^^b^
Humor-HSQ with No-Humor HSQ	0.33^*^	0.38^*^	0.48^*^	−0.15^*^
**ITEM 8**				
HSQ with Humor-HSQ	0.77^*^	0.27^*^^a^	0.14^a^	0.57^*^
HSQ with No-Humor HSQ	0.71^*^	0.71^*^^b^	0.35^*^^b^	0.61^*^
Humor-HSQ with No-Humor HSQ	0.54^*^	−0.01	0.04	0.32^*^

*N = 187. AF, affiliative; SE, self-enhancing; AG, aggressive; SD, self-defeating*.

* *p < 0.05*.

a, b *Correlations with different superscripts differed significantly from one another (at the 0.05 level)*.

As shown in Table 2, six of eight items (all except for items 9 and 29) of the HSQ affiliative scale correlated significantly higher with the homologous items of the Humor-HSQ than with the No-Humor-HSQ. For the HSQ self-enhancing scale, two items (items 6 and 22) correlated significantly higher with the Humor-HSQ than with the No-Humor-HSQ, while this effect was reversed for two other items (items 26 and 30). For the HSQ aggressive and self-defeating scales, four (items 11, 19, 27, and 31) and three items (items 20, 24, and 28), respectively, showed significantly different correlations, indicating that their relationship with the No-Humor-HSQ was significantly higher than the relationship with the Humor-HSQ.

### Discussion

The aim of Study 1 was to test the construct validity of the HSQ by comparing the original HSQ with newly created Humor- and No-Humor-HSQ versions. Construct validity would be supported if the humor content turned out to be more important than the no-humor elements, evidenced by predicable patterns of internal consistencies and intercorrelations. First, the expected pattern of internal consistencies was found (Humor-HSQ scales > HSQ scales > No-Humor-HSQ). Thus, removing construct-irrelevant context made the four scales more similar, and removing the construct-relevant content made them less similar. Interestingly, the No-Humor-HSQ scales mostly had acceptable internal consistencies (McDonald's omega > 0.70, except for the aggressive scale with 0.42), indicating that participants answered the no-humor elements within each HSQ scale somewhat similarly. That is, the no-humor elements within the HSQ items did not average out at the scale-level and were thus able to contribute reliable variance to the No-Humor-HSQ scales.

Second, the pattern of intercorrelations of the affiliative and self-enhancing scales supported the primary importance of the humor core in two HSQ scales. Specifically, the intercorrelation between the HSQ and the Humor-HSQ was significantly higher than the one between the HSQ and the No-Humor-HSQ. The self-defeating scale showed the reverse effect, with the HSQ being more similar to the No-Humor- (*r*^2^ = 0.58) than the Humor-HSQ (*r*^2^ = 0.37). In other words, the non-humorous elements (i.e., construct-irrelevant variance) were more important than the humor core (i.e., construct-relevant variance) in the HSQ self-defeating scale.

The pattern found in the observed correlations was also corroborated in the true-score correlations. The HSQ affiliative scale was virtually identical with the Humor-HSQ scale, supporting the interpretation that it is mainly determined by humor. This was also the case for the individual items, yielding strong support for the construct validity of the HSQ affiliative scale. Along these lines, the HSQ affiliative scale correlated positively with all Humor-HSQ scales (large effects), suggesting that the humor contents of the four scales resembled the affiliative humor style, that is, amusing others, liking to laugh, and making jokes.

The true-score correlations showed that HSQ self-enhancing scale was highly similar to the Humor-HSQ scale and the No-Humor HSQ scale. Interestingly, these effects largely varied across the eight self-enhancing items. Item 6 (“Even when I'm by myself, I'm often amused by the absurdities of life.”) and Item 22 (“If I am feeling sad or upset, I usually lose my sense of humor”) showed higher correlations to the Humor-HSQ than the No-Humor-HSQ; that is, humor was more relevant in these two items than the context. Thus, people who are more or less frequently amused by the incongruities of life and who keep or lose their sense of humor seem to do so independent of the social context or the emotional states they are in. By contrast, Item 26 (“It is my experience that thinking about some amusing aspect of a situation is often a very effective way of coping with problems”) and Item 30 (“I don't need to be with other people to feel amused—I can usually find things to laugh about even when I'm by myself”) showed higher correlations to the No-Humor-HSQ than the Humor-HSQ. Thus, the context in these items was more relevant than the humor. This implicates that either the context is dominant in the items (i.e., coping with problems or being by oneself), or humor is not a determining or unique factor in such situations (e.g., people cope with problems humorously, but also by non-humorous means). Thus, the construct validity of the HSQ self-enhancing scale can be mostly supported, though two of the eight items were largely determined by construct-irrelevant variance.

The true-score correlations of the HSQ aggressive scale with the homologous Humor-HSQ and No-Humor-HSQ scales were 1.00, showing that the HSQ scale was identical to both experimentally manipulated versions. Note that the latter true-score correlation exceeded 1.00 in the computation, indicating an overcorrection due to the low internal consistency of the No-Humor-HSQ aggressive scale (see Muchinsky, 1996). However, the relevance of the No-Humor-HSQ was also supported in the observed correlations and in the item-level analyses: Four of the eight HSQ aggressive items showed significantly higher correlations to the No-Humor-HSQ than to the Humor-HSQ. These effects were most pronounced for Item 11 (“When telling jokes or saying funny things, I am usually not very concerned about how other people are taking it”) and Item 19 (“Sometimes I think of something that is so funny that I can't stop myself from saying it, even if it is not appropriate for the situation”). Again, this yields two possible interpretations: Either the context is dominant (not caring about others opinions or feelings, and acting impulsively and inappropriately) or humor is not a decisive factor in these items (e.g., people saying something humorous and non-humorous while not being concerned about others, or doing so impulsively and inappropriately). Thus, the construct validity of the HSQ aggressive can be partly supported, yet the strong context effects found in specific items require further scrutiny.

In contrast to the other HSQ scales, the HSQ self-defeating scale was almost identical to the homologous No-Humor-HSQ scale, but not to the Humor-HSQ scale. This effect was also found in three of the eight HSQ self-defeating items: Item 5 (“I often go overboard in putting myself down when I am making jokes or trying to be funny”), Item 6 (“When I am with friends or family, I often seem to be the one that other people make fun of or joke about”) and Item 7 (“If I am having problems or feeling unhappy, I often cover it up by joking around, so that even my closest friends don't know how I really feel”). Again, either the humor in the items might not be very salient (and thus non-humorous, but similar behaviors strongly overlap with the item), or the context is dominating (e.g., going overboard, or covering up problems and negative feelings). Additionally, the self-defeating humor core was compatible with all humor styles (also with the self-enhancing one).

These findings might rather support the interpretation that the context was dominating in the HSQ self-defeating items. This suggests a potentially impactful implication: Probably the humor content can be meaningfully interpreted, yet not along the lines of the self-defeating humor style as proposed by Martin et al. (2003). This could potentially explain the contradiction between the conception of the HSQ self-defeating scale as mostly maladaptive (Martin et al., 2003), and the humor core of laughing at oneself, which is generally considered a positive trait (e.g., McGhee, 1999; see also Ruch and Heintz, 2013). The negative aspect of the HSQ self-defeating scale could be due to the primary influence of the non-humor elements of this scale, which are mostly negative connoted (like putting oneself down excessively).

Taking a closer look at the pattern of intercorrelations within one HSQ version also revealed that all HSQ-Humor scales correlated significantly and positively with one another (medium to large effects), while this was not the case for the HSQ and No-Humor-HSQ scales. The latter two HSQ-versions also had negative scale intercorrelations. Hence, the participants rated the humor contents in the four scales quite similarly, while they differentiated the scales better once the non-humorous elements were involved. This underlines that the differentiation between the four HSQ scales might be more driven by varying their non-humorous elements *across* the scales (e.g., being with others vs. being alone, being in a sad or depressed mood vs. being cheerful) than by their humor cores.

Besides testing the construct validity, separating the construct-relevant and construct-irrelevant elements also allows for testing their contributions to correlations with other constructs and outcomes (i.e., criterion validity). For example, it was shown that correlations of the HSQ with personality traits and aspects of psychological well-being were mainly driven by the No-Humor-HSQ, while relations to other humor constructs (such as laughing at oneself) were mainly driven by the Humor-HSQ (Ruch and Heintz, 2013). This effect was most pronounced for the self-defeating scale, which is in line with the present findings. Study 2 aims at investigating the relevance of the construct-irrelevant context in the scales in relation to several criteria (personality and well-being), replicating and extending these previous findings.

## Study 2: criterion validity of the humor in the humor styles questionnaire

In addition to construct validity, it is relevant to investigate the criterion validity of the HSQ scales. The relevant criteria of the HSQ are humor and psychosocial well-being, as the humor style concepts were derived from the literature in these two areas, and as the humor styles are defined as everyday functions of humor that are relevant to psychosocial well-being (Martin et al., 2003). Besides relating the HSQ to humor-related scales (e.g., Martin et al., 2003; Kuiper et al., 2004; Ruch and Heintz, 2016) and humor behaviors (Heintz, 2017), the HSQ is usually compared to personality traits (for a meta-analysis with the Big Five personality traits, see Mendiburo-Seguel et al., 2015) and to subjective well-being (e.g., Edwards and Martin, 2010, 2014; Jovanovic, 2011; Ruch and Heintz, 2013; Maiolino and Kuiper, 2014). These relationships have usually been associated with the humor in the HSQ scales. However, previous studies found rather low incremental validities of the HSQ scales in explaining subjective well-being (Jovanovic, 2011; Dyck and Holtzman, 2013; Ruch and Heintz, 2013; Maiolino and Kuiper, 2014; Heintz, 2017). Also the results from Study 1 cast doubt on the role of humor in the HSQ self-defeating scale, making further investigations on the criterion validity in terms of personality and well-being necessary.

Study 2 investigates the criterion validity of the HSQ scales with the Big Five personality traits, namely extraversion, agreeableness, conscientiousness, emotional stability, and culture (also labeled openness or intellect), and subjective well-being, consisting of life satisfaction as a cognitive component and positive and negative affect as affective components. In line with previous findings positive relationships are expected for the affiliative and self-enhancing scales with extraversion and openness to experience/culture. The self-enhancing scale should also positively correlate with emotional stability and with agreeableness. The aggressive scale should correlate negatively with conscientiousness and agreeableness. The self-defeating scale should correlate negatively with emotional stability and conscientiousness. In terms of subjective well-being, the affiliative and self-enhancing scales should correlate positively with life satisfaction and positive affect, and negatively with negative affect, while this pattern should be reversed for the self-defeating scale. No significant correlations are expected for the aggressive scale. The change in this relationship once the homologous No-Humor-HSQ scales are controlled for is utilized as an indicator of the criterion validity of the HSQ scales.

One previous study employed the same approach to investigate the criterion validity of the HSQ in terms of six indicators of psychological well-being (Ruch and Heintz, 2013). They found that only 3 of the 13 significant relationships remained significant once the No-Humor-HSQ was taken into account. This approach was also employed in Study 2, instead of investigating the Humor-HSQ scales directly, as the Humor-HSQ still contains some elements that are not related to humor, simply because the items needed to be meaningful by themselves (e.g., “I let others laugh at me, which keeps them in in good spirits.” for self-defeating or “I usually try to think of something funny about a situation.” for self-enhancing). The No-Humor-HSQ, by contrast, is parallel to the HSQ, and the only difference lies in the absence vs. presence humor-related terms and phrases. The test of criterion validity conducted in Study 2 is thus stricter, but also more precise. Based on the previous findings on the incremental validity and criterion validity of the HSQ scales and Study 1, we expected small criterion validities of the HSQ beyond the No-Humor-HSQ.

### Materials and methods

#### Participants

Of the 474 German-speaking participants that started the survey, 272 (57.4%) completed all the items. A total of 261 participants (30.7% men) with a median age of 24.00 (*M* = 27.26, *SD* = 10.11) ranging from 18 to 69 years provided valid responses in this study (participants were excluded if they indicated an age below 18 years [*n* = 9] or if they showed aberrant answer patterns like always using the same answer option or answering randomly [*n* = 2]). Participants were primarily Swiss (63.2%), German (26.8%), and from several other nations. Most participants were well-educated, with 50.2% being college or university students, 23.0% having passed tertiary education, 22.2% having A-levels, and 4.6% having <12 years of education. A subsample of the present data was used by Heintz (2017). None of the present results have been published before, and they extend the previous study by investigating the cross-sectional correlations among the HSQ, the No-Humor HSQ, personality, and subjective well-being.

#### Instruments

##### Humor styles questionnaire (HSQ; Martin et al., 2003; German version by Ruch and Heintz, 2016)

The same version of the HSQ was used as in Study 1.

##### Context version derived from the HSQ (no-humor-HSQ)

The same version of the No-Humor-HSQ was used as in Study 1.

##### MRS-25

*Inventory of minimally redundant scales (MRS; Schallberger and Venetz, 1999)*. The MRS employs bipolar adjectives to assess the Big Five personality traits extraversion (e.g., talkative/quiet), agreeableness (e.g., well-tempered/short-tempered), conscientiousness (e.g., organized/disorganized), emotional stability (e.g., relaxed/oversensitive), and culture (e.g., artistic/inartistic). The 25-item version was used (five items for each trait). It employs a six-point Likert scale with mirrored labels: “very” (−3/+3), “quite” (−2/+2), and “rather” (−1/+1).

##### SWLS

*Satisfaction with life scale (SWLS; Diener et al., 1985)*. The SWLS measures life satisfaction (e.g., “I am satisfied with my life”) with five items. It employs a seven-point Likert scale from “strongly disagree” (1) to “strongly agree” (7).

##### PANAS

*Positive and negative affect schedule (PANAS; Watson et al., 1988)*. The PANAS measures positive affect (e.g., enthusiastic) and negative affect (e.g., nervous) with 10 items each. It employs a five-point Likert scale from “very slightly or not at all” (1) to “extremely” (5).

#### Procedure

The data were collected in an online survey (www.unipark.info) using the German versions of the instruments. The order of presentation was PANAS, No-Humor-HSQ, SWLS, a humor questionnaire (not relevant for the present study), MRS-25, and HSQ. All items were obligatory to answer. Participants were recruited in similar venues as those of Study 1. They were offered a personalized feedback and/or course credit in psychology for their participation. The study was conducted in compliance with the local ethical guidelines and participants provided their online informed consent.

#### Data analysis

As in Study 1, internal consistencies (McDonald's omega) and scale intercorrelations were computed to compare the HSQ and the No-Humor-HSQ. Criterion validity was investigated in stepwise multiple regression, entering each No-Humor-HSQ scale in the first step and the homologous HSQ scale in the second step. Multicollinearity in the regression was low (variance inflation factors between 1.8 and 2.5).

### Results

Table 3 shows the descriptive statistics, internal consistencies, and correlations with the HSQ scales and the No-Humor-HSQ scales. Replicating the findings of Study 1, the HSQ scales were always (numerically) more internally consistent than the corresponding No-Humor-HSQ scales. The No-Humor-HSQ scales again showed high internal consistencies (>0.60), this time also for the aggressive scale. Correlations between the homologous scales were high and comparable to Study 1. The true-score correlations supported the equivalence of the HSQ and No-Humor-HSQ scales for self-enhancing (0.95), aggressive (1.00), and self-defeating (1.00), but not for affiliative (0.80), again replicating the findings from Study 1. In addition, the correlations between the HSQ items and the homologous No-Humor-HSQ items (shown in Table 4) were highly similar to Study 1.

**Table 3:** **Descriptive statistics, internal consistencies, and correlations with the Humor Styles Questionnaire (HSQ) scales and the No-Humor-HSQ Scales**.

	***M***	***SD***	**ω**	**Correlations with the HSQ**				**Correlations with the No-Humor-HSQ**			
				**AF**	**SE**	**AG**	**SD**	**AF**	**SE**	**AG**	**SD**
**HSQ**											
Affiliative^a^	42.62	7.99	0.88								
Self-enhancing^a^	36.87	8.11	0.84	0.42^*^							
Aggressive^a^	27.47	6.78	0.70	0.15^*^	0.04						
Self-defeating^a^	24.95	8.16	0.82	0.01	0.04	0.00					
**NO-HUMOR-HSQ**											
Affiliative^a^	38.89	8.29	0.83	0.68^*^	0.29^*^	0.13^*^	−0.12				
Self-enhancing^a^	36.06	6.73	0.70	0.23^*^	0.73^*^	−0.11	−0.06	0.28^*^			
Aggressive^a^	27.74	6.35	0.61	0.01	−0.07	0.75^*^	−0.07	0.18^*^	−0.14^*^		
Self-defeating^a^	24.72	7.28	0.71	−0.15^*^	−0.13^*^	0.03	0.77^*^	−0.27^*^	−0.21^*^	0.02	
**PERSONALITY**											
Agreeableness^b^	4.51	0.73	0.73	0.23^*^	0.32^*^	−0.44^*^	0.02	0.10	0.32^*^	−0.54^*^	−0.10
Conscientiousness^b^	4.29	0.94	0.87	0.00	0.01	−0.24^*^	−0.08	0.06	0.08	−0.19^*^	−0.14
Emotional stability^b^	3.76	1.01	0.86	0.33^*^	0.40^*^	0.12	−0.30^*^	0.48^*^	0.47^*^	0.13^*^	−0.48^*^
Extraversion^b^	4.10	1.05	0.87	0.58^*^	0.26^*^	0.03	−0.06	0.72^*^	0.21^*^	0.09	−0.22^*^
Culture^b^	4.46	0.87	0.87	0.29^*^	0.28^*^	−0.13^*^	−0.05	0.34^*^	0.24^*^	−0.10	−0.07
**SUBJECTIVE WELL-BEING**											
Positive affect^c^	33.42	6.12	0.85	0.25^*^	0.33^*^	0.00	−0.20^*^	0.43^*^	0.40^*^	0.08	−0.32^*^
Negative affect^c^	19.74	7.03	0.89	−0.20^*^	−0.35^*^	−0.04	0.34^*^	−0.32^*^	−0.40^*^	0.01	0.42^*^
Life satisfaction^d^	4.45	1.49	0.91	0.28^*^	0.34^*^	0.14^*^	−0.35^*^	0.48^*^	0.46^*^	0.15^*^	−0.49^*^

*N = 261. ω = McDonald's omega*.

* *p < 0.05*.

a *Theoretical minimum = 8, maximum = 56*.

b *Theoretical minimum = 1, maximum = 6*.

c *Theoretical minimum = 10, maximum = 50*.

d *Theoretical minimum = 1, maximum = 7*.

**Table 4:** **Intercorrelations of the Humor Styles Questionnaire (HSQ) items with the Homologous items of the No-Humor-HSQ**.

	**AF**	**SE**	**AG**	**SD**
Item 1	0.38^*^	0.50^*^	0.45^*^	0.51^*^
Item 2	0.44^*^	0.14^*^	0.54^*^	0.55^*^
Item 3	0.43^*^	0.60^*^	0.60^*^	0.65^*^
Item 4	0.59^*^	0.70^*^	0.44^*^	0.15^*^
Item 5	0.40^*^	0.68^*^	0.77^*^	0.62^*^
Item 6	0.43^*^	0.40^*^	0.56^*^	0.67^*^
Item 7	0.41^*^	0.59^*^	0.67^*^	0.78^*^
Item 8	0.76^*^	0.61^*^	0.35^*^	0.47^*^

*N = 261. AF, affiliative; SE, self-enhancing; AG, aggressive; SD, self-defeating*.

* *p < 0.05*.

#### Relationships with personality and subjective well-being

As shown in Table 3, both the HSQ and the No-Humor-HSQ scales showed similar and mostly significant relationships to personality and subjective well-being: Affiliative related most strongly to extraversion, self-enhancing to emotional stability, aggressive to lower agreeableness, and self-defeating to lower emotional stability. The relationships were in general similar to the ones reported in the meta-analysis by Mendiburo-Seguel et al. (2015). Also in line with previous findings, affiliative, and self-defeating correlated positively with subjective well-being, while self-defeating was negatively related to it.

#### Criterion validity beyond context

Next, the criterion validity of the HSQ over and above its construct-irrelevant context is investigated, yielding information on the specific relationships of the humor in the HSQ (as construct-relevant content). Table 5 provides the results of standard multiple regression analyses explaining subjective well-being with the No-Humor-HSQ in step 1 and the HSQ in step 2 (separately for each humor style).

**Table 5:** **Stepwise multiple regression analyses predicting subjective well-being with the No-Humor (NH) version of the Humor Styles Questionnaire (HSQ) in step 1 and the HSQ in step 2 (separately for each scale; only last step reported)**.

	**Big Five personality traits**										**Subjective well-being**					
	**A**		**C**		**ES**		**Extraversion**		**Culture**		**PA**		**NA**		**LS**	
**Predictor**	**Δ*R*^2^**	**β**	**Δ*R*^2^**	**β**	**Δ*R*^2^**	**β**	**Δ*R*^2^**	**β**	**Δ*R*^2^**	**β**	**Δ*R*^2^**	**β**	**Δ*R*^2^**	**β**	**Δ*R*^2^**	**β**
Step 1	0.01		0.00		0.23^*^		0.52^*^		0.11^*^		0.19^*^		0.10^*^		0.23^*^	
NH-HSQ AF		−0.11		0.12		0.48^*^		0.61^*^		0.26^*^		0.43^*^		−0.32^*^		0.48^*^
Step 2	0.05^*^		0.00		0.00		0.01^*^		0.01		0.00		0.00		0.01	
HSQ AF		0.30^*^		−0.08		0.01		0.16^*^		0.11		−0.09		0.03		−0.10
Total *R*^2^	0.06^*^		0.01		0.23^*^		0.53^*^		0.12^*^		0.19^*^		0.10^*^		0.24^*^	
Step 1	0.10^*^		0.01		0.22^*^		0.04^*^		0.06^*^		0.16^*^		0.16^*^		0.22^*^	
NH-HSQ SE		0.20^*^		0.15		0.39^*^		0.04		0.08		0.40^*^		−0.40^*^		0.46^*^
Step 2	0.01		0.01		0.01		0.03^*^		0.02^*^		0.00		0.01		0.00	
HSQ SE		0.17		−0.10		0.11		0.24^*^		0.22^*^		0.07		−0.12		−0.01
Total *R*^2^	0.12^*^		0.01		0.23^*^		0.07^*^		0.08^*^		0.16^*^		0.17^*^		0.22^*^	
Step 1	0.29^*^		0.04^*^		0.02^*^		0.01		0.01		0.01		0.00		0.02^*^	
NH-HSQ AG		−0.48^*^		−0.02		0.08		0.15		−0.01		0.08		0.01		0.15^*^
Step 2	0.00		0.02^*^		0.00		0.00		0.01		0.01		0.00		0.00	
HSQ AG		−0.08		−0.23^*^		0.06		−0.08		−0.12		−0.16		−0.09		0.06
Total *R*^2^	0.29^*^		0.06^*^		0.02		0.01		0.02		0.02		0.00		0.02^*^	
Step 1	0.01		0.02^*^		0.23^*^		0.05^*^		0.01		0.10^*^		0.18^*^		0.24^*^	
NH-HSQ SD		−0.28^*^		−0.19^*^		−0.59^*^		−0.41^*^		−0.08		−0.32^*^		0.42^*^		−0.49^*^
Step 2	0.02^*^		0.00		0.01		0.03^*^		0.00		0.01		0.00		0.00	
HSQ SD		0.24^*^		0.07		0.15		0.25^*^		0.01		0.11		0.04		0.07
Total *R*^2^	0.03^*^		0.02		0.24^*^		0.07^*^		0.01		0.11^*^		0.18^*^		0.24^*^	

*N = 261. AF, affiliative; SE, self-enhancing; AG, aggressive; SD, self-defeating; A, agreeableness; C, conscientiousness; ES, emotional stability; PA, positive affect; NA, negative affect; LS, life satisfaction*.

* *p < 0.05*.

As shown in Table 5, the variance that the HSQ scales explained over and above their homologous No-Humor-HSQ scales in subjective well-being was not significant. Thus, the humorous contents in the HSQ did not uniquely explain subjective well-being once the context elements were controlled for (although 10 significant correlations were originally present). The magnitude of the effects was comparable to the previous study (Ruch and Heintz, 2013). In terms of personality, seven regressions yielded significant amounts of explained variance (1.0–5.0%) for the HSQ scales (from 12 originally significant correlations). The humor in the HSQ affiliative scale was uniquely related to agreeableness and extraversion, and the humor in the HSQ self-enhancing scale was uniquely related to extraversion and openness. The humor in the HSQ aggressive scale showed a unique negative relationship to conscientiousness, and the humor in the HSQ self-defeating scale showed unique relationships to agreeableness and extraversion. Thus, while no significant criterion validities were found between the humor in the HSQ scales and subjective well-being, each HSQ scale had their unique pattern of criterion validities across the Big Five personality traits.

### Discussion

Study 2 aimed at partially replicating the findings of Study 1 regarding the construct validity of the HSQ and at extending the validity analyses to the criterion validity in terms of personality and subjective well-being. The relationships between the HSQ scales and the No-Humor-HSQ scales were highly similar to Study 1, thus replicating and strengthening the previous findings on the construct validity of the HSQ.

Criterion validities varied across the two sets of criteria (personality and subjective well-being). Seven of the 12 relationships between the HSQ and the Big Five personality traits were robust beyond the No-Humor-HSQ. Thus, the humor in each of the four humor styles had a unique relevance to one or two personality traits. The humor in the HSQ affiliative scale was relevant to agreeableness and extraversion, showing that it comprised unique prosocial and social qualities. The humor in the HSQ self-enhancing scale was relevant to extraversion and culture, also supporting a unique social quality, but also a cognitive aspect. The latter might be due to recognizing incongruities in one's surroundings and being amused by them, which is a core component of humor. Openness (or culture) has thus also been implied in appreciating non-sense humor and in humor creation (e.g., Galloway and Chirico, 2008; Nusbaum, 2015). However, the relationship of the HSQ self-enhancing scale to emotional stability was not specific to humor, showing that enhancing oneself and coping with problems could be achieved non-humorously.

The humor in the HSQ aggressive scale uniquely related to lower conscientiousness (but not agreeableness). Thus, aggressive humor did not have an antisocial quality, suggesting that the label “aggressive” might not fit well to the humor content of this scale. The relationship to lower conscientiousness could probably be explained by a playful attitude underlying this humor style (e.g., teasing and making fun of others, but not in a hurtful or malicious way). This interpretation would be supported by previous studies relating the HSQ aggressive scale to lower seriousness (Martin et al., 2003) and the finding that the Humor-HSQ aggressive scale correlated positively with playfulness (Ruch and Heintz, 2013). The humor in the HSQ self-defeating scale uniquely related to agreeableness and extraversion. Thus, although the HSQ self-defeating was unrelated to both personality traits, the humor in this scale had a unique prosocial and social quality, similar to the humor in the affiliative scale. As with self-enhancing, no humor-specific effects were present for emotional stability, clearly limiting the maladaptive interpretation of self-defeating humor.

For subjective well-being, the criterion validity of the HSQ cannot be supported, as no significant amounts of variance could be explained by the HSQ scales once their homologous No-Humor-HSQ scales were controlled for. Thus, the frequently found relationships between the HSQ and subjective well-being (positively for affiliative and self-enhancing and negative for self-defeating) seem to be driven mostly or entirely by the non-humorous elements (i.e., the construct-irrelevant context) and not the humor itself (i.e., construct-relevant content). This is also in line with the usually low incremental validities of the HSQ scales in explaining subjective well-being over and above the Big Five personality traits (Jovanovic, 2011; Dyck and Holtzman, 2013; Ruch and Heintz, 2013).

Two implications can be derived from the present findings: First, humor was not a decisive factor in the relationships between the HSQ and subjective well-being. For example, it cannot be firmly concluded that affiliative and self-enhancing humor is positive and that self-defeating humor is negative. Instead, the non-humorous elements in these humor styles (e.g., liking to be with others, being able to cope with problems, or putting oneself down excessively) were the active ingredients in the relationship with subjective well-being. Second, which aspects of these non-humorous elements is most relevant in this relationship (e.g., situations, functions, states, or evaluations or combination or interaction between them) remains open for further investigation.

Does this mean that the humor in the HSQ is completely irrelevant to subjective well-being? As stated before, the present test is a rather strict one. Directly correlating the Humor-HSQ scales to six aspects of psychological well-being revealed positive correlations for affiliative and self-enhancing humor, but zero correlations for self-defeating humor (Ruch and Heintz, 2013). In a similar vein, daily-measured humor behaviors that were similar (but not equivalent) to the Humor-HSQ scales exhibited incremental validity in explaining subjective well-being beyond personality and the HSQ (Heintz, 2017); specifically cheerful (similar to affiliative), amused (similar to self-enhancing), and self-directed (similar to self-defeating) humor behaviors. Thus, there is evidence that the humor in the HSQ can be positive in terms of psychological well-being. Most importantly, the negativity of the HSQ self-defeating scale was not supported in these less stringent analyses. This humor style can thus best be interpreted as having a negative context, yet the humor in it is either unrelated to psychological well-being or positive. This precludes drawing conclusions such as “learning how to decrease one's use of self-defeating humor” (Maiolino and Kuiper, 2014, p. 568) for enhancing one's well-being. The conclusion should rather be “putting oneself less down” (whether with humor or not) to increase one's well-being, which seems to be both a trivial and circular reasoning.

## General discussion

Study 1 and 2 yielded converging evidence that the construct validity of the HSQ affiliative scale can be fully supported, while the construct validities of the HSQ self-enhancing and aggressive scales yielded mixed findings. The construct validity of the HSQ self-defeating scale could not be supported. Thus, the term “humor” in the humor styles seems appropriate for affiliative, needs be used with caution for self-enhancing and aggressive, and seems inappropriate for self-defeating. Combining these findings with the criterion validities, the humor content in the self-enhancing humor style might be rather labeled cultured or open-minded affiliative humor, and the humor in the aggressive humor style might rather be playful teasing.

The lack of criterion validity in terms of subjective well-being necessitates a reinterpretation of the role that humor plays in subjective well-being. For the affiliative and the self-enhancing humor style, the extent to which they are relevant to subjective well-being might have been overestimated in previous studies, as the primary motor of the relationships seems to lie in the non-humorous elements (e.g., the “style,” or function, or contexts). While this only affects the magnitude of the relationships, the consequences are more severe for the HSQ self-defeating scale: This humor style has been implied to be negative, yet both its construct and criterion validities showed that the non-humorous elements determined this humor style more than humor did, and no negative—but rather positive—effects emerged. Importantly, this was also the case when less stringent tests were used; that is, when the humor in the self-defeating humor style were directly related to well-being (Ruch and Heintz, 2013; Heintz, 2017). Thus, the humor in the self-defeating humor style might be quite similar to the notion of an adaptive ability of laughing at yourself (McGhee, 1999) after all.

While the present study focused on one instrument of relevance for humor research, the general principle is independent of the instrument studied. Indeed, we believe that the methodology and considerations used here can be applied to psychological questionnaires in general, and in particular when the items are more complex and merge core behaviors and contextual variables. This is often the case, as traits are defined by behaviors that are consistent across time and situations. This is usually implemented by varying the context in which the behaviors occur and a strong context might generate variance itself. Also items may contain conditions for behaviors, where the conditions already have different probabilities, and hence contribute to the variance in response to the item. For example, an item “when traveling abroad, I usually prefer to stay away from problem areas” might be envisioned to be an item for prudence. However, very prudent people might disagree to the item when they just do not travel abroad at all. This made-up item demonstrates that only some of the variance is due to prudence, but the other part of the variance is actually capturing the opposite of it. Thus, the importance of item wording should not be underestimated, and it is best already considered during the process of test construction. Cognitive interviewing techniques (see e.g., Willis, 2004), for example, can detect whether items are understood in way that is intended by the creator.

### Limitations and suggestions for future research

First, the generalization of the results is limited to a German-speaking, young, and well-educated sample; hence, replications in other languages, cultures, and samples with a wider range in age or education are desirable. Second, the order of presentation of the different HSQ versions was not randomized, and thus any systematic influences associated with the order of presentation could have interfered with our findings. Third, the present study focused on one of two construct-relevant contents in the humor styles, namely humor (which could certainly considered to be the more prominent one given the name of the construct and their treatment in previous research). Investigating the role of “style” (as functions or uses) in the composition of the HSQ and its role in the relationships with other criteria would complement the present investigations of construct and criterion validity. Fourth, further experimental evidence is necessary to investigate the causal relationships between the HSQ, humor, personality and subjective well-being. For example, investigating which emotional states are associated and elicited by self-defeating humor experiences, or by self-defeating humor trainings, would enhance our understanding of the role that the humor entailed in the HSQ plays in criteria such as subjective well-being. Fifth, our investigations of the criterion validity of the HSQ scales focused on personality and subjective well-being. As the HSQ has been frequently studied in relation to other trait-like variables (such as character strengths; Edwards and Martin, 2014), extending the scope to further criteria would yield a more complete picture of the role that the humor in the humor styles plays.

## Conclusion

The present studies showed that humor might not be as relevant in the humor styles as would be naturally and usually assumed. This might explain why Martin et al. (2003) found that “the HSQ accounts for a greater proportion of variance in well-being than do several existing self-report humor scales.” (p. 72 f.), which was also corroborated by Edwards and Martin (2014). If humor measures are compared to a measure that contains a large proportion of non-humorous elements that are related to well-being, the latter instrument might seem “better”—yet this does not tell us anything new about the relevance of humor in well-being. Thus, Martin et al.'s (2003) outlook that research with the HSQ “may provide better understanding of the ways in which humor may function as an adaptive resource for psychological health, as well as the ways in which it may interfere with healthy adjustment and impair relationships with others.” (p. 73) seems to be hard to fulfill with the HSQ (at least in its current form). Researchers interested the relationships of humor to subjective well-being and potentially other well-being outcomes should thus be cautioned, as the HSQ scales yield rather limited information on the role that humor itself plays in these relationships (and in the case of self-defeating humor potentially misleading information). Other approaches to humor styles, such as the Humor-Behavior Q-Sort Deck (Craik et al., 1996) or comic styles (e.g., Schmidt-Hidding, 1963) might be fruitful alternatives in this regard. Future research might yield smaller, yet likely more realistic relationships, between humor and well-being.

## Ethics statement

These studies were carried out in accordance with the recommendations of the Ethical Principles of Psychologists and Code of Conduct (APA) and the Ethical Guidelines for Psychologists of the Swiss Psychological Society (SGP), as outlined by the ethics committee of the Faculty of Arts at the University of Zurich, with online informed consent from all subjects. All subjects gave online informed consent in accordance with the Declaration of Helsinki. It was not possible to obtain written informed consent, as both studies were conducted solely via the Internet, yet online informed consent was shown to be similar to written informed consent (see Varnhagen et al., 2005). The protocol was exempt from approval as stated by the guidelines of the ethics committee of the Faculty of Arts at the University of Zurich, as it passed a checklist of ethical innocuousness (which serves as ethical approval in accordance with the local guidelines).

## Author contributions

All authors listed have made substantial, direct and intellectual contribution to the work, and approved it for publication.

### Conflict of interest statement

The authors declare that the research was conducted in the absence of any commercial or financial relationships that could be construed as a potential conflict of interest.

## Footnotes

^1^Control analyses showed that none of the results were altered by excluding these participants.

## Appendix

**Table A1:** **Overview of the 32 items of the humor- and no-humor versions of the Humor Styles Questionnaire**.

**Scale**	**Item**	**No-Humor-HSQ**	**Humor-HSQ**
AF	1	I usually don't talk or converse much with other people. [recoded]	I usually don't laugh or joke around much. [recoded]
SE	2	If I am feeling depressed, I can usually put myself in a better mood with something beautiful.	I can usually cheer myself up with humor.
AG	3	If someone makes a mistake, I will often reproach them about it.	I often tease others.
SD	4	I let people offend me or look down one me more than I should.	I let people laugh at me or make fun at my expense.
AF	5	I don't have to work very hard at impressing other people—I seem to be a naturally influencing person.	I make others laugh easily—I am a humorous person.
SE	6	Even when I'm by myself, I often occupy myself with the little things in life.	I'm often amused by the absurdities of life.
AG	7	People are never offended or hurt by my manner of speaking. [recoded]	My sense of humor is never offending or hurting. [recoded]
SD	8	I will often get carried away in putting myself down if it makes my family or friends feel good.	I often put myself down and thus make others laugh.
AF	9	I rarely impress other people by telling enthralling stories about myself. [recoded]	I rarely tell funny stories about myself, which make others laugh. [recoded]
SE	10	If I am feeling upset or unhappy, I usually try to think of something beautiful about the situation to make myself feel better.	I usually try to think of something funny about a situation.
AG	11	When telling experiences or saying enthralling things, I am usually not very concerned about how other people are taking it.	I usually tell jokes or say funny things.
SD	12	I often try to make people like or accept me more by saying something appealing about my own weaknesses, misapprehensions, or faults.	I often say something funny about my own weaknesses, blunders, or faults.
AF	13	I talk or converse a lot with my friends.	I laugh and joke a lot.
SE	14	My positive outlook on life keeps me from getting overly upset or depressed about things.	I have a humorous outlook on life.
AG	15	I do not like it when people tell experiences or say things as a way of criticizing or putting someone down. [recoded]	I do not like criticizing or putting-down humor. [recoded]
SD	16	I don't often say enthralling things to put myself down. [recoded]	I rarely say funny things about myself. [recoded]
AF	17	I usually don't like to tell experiences or to impress people. [recoded]	I usually don't tell jokes or amuse people. [recoded]
SE	18	If I'm by myself and I'm feeling unhappy, I make an effort to think of something beautiful to make me feel better.	I always think of something funny to cheer myself up.
AG	19	Sometimes I think of something that is so enthralling that I can't stop myself from saying it, even if it is not appropriate for the situation.	Sometimes I think of extremely funny things.
SD	20	I often go overboard in putting myself down when I am telling experiences or trying to be communicative.	I often make jokes about myself or make fun of myself.
AF	21	I enjoy impressing people.	I make people laugh.
SE	22	If I am feeling sad or upset, I usually lose my serenity. [recoded]	I never lose my sense of humor.
AG	23	I never participate in offending others even if all my friends are doing it. [recoded]	I never laugh at others. [recoded]
SD	24	When I am with friends or family, I often seem to be the one that other people look down on or offend.	I let others often make fun of me or joke about me.
AF	25	I don't often converse with my friends. [recoded]	I don't often joke around. [recoded]
SE	26	It is my experience that thinking about some beautiful aspect of a situation is often a very effective way of coping with problems.	I often think about some amusing aspect of a situation.
AG	27	If I don't like someone, I often criticize or reproach them to put them down.	I often use humor about others or tease them.
SD	28	If I am having problems or feeling unhappy, I often cover it up by telling an experience, so that even my closest friends don't know how I really feel.	I often joke around.
AF	29	I usually can't think of enthralling things to say when I'm with other people. [recoded]	I usually can't think of witty things. [recoded]
SE	30	I don't need to be with other people to feel good—I can usually find things to occupy myself with even when I'm by myself.	I am usually amused and I can find things to laugh about.
AG	31	Even if something is really relevant to me, I will not say anything or criticize it if someone will be offended. [recoded]	I always laugh or joke about something that is really funny to me.
SD	32	Letting others offend me is my way of making my friends and family feel good.	I let others laugh at me, which keeps them in in good spirits.

*AF, affiliative; SE, self-enhancing; AG, aggressive; SD, self-defeating. The order of the items and the response options are the same as in the HSQ (Martin et al., 2003)*.
